# Supplementary material for: LreEF1A4, a Translation Elongation Factor from Lilium regale, Is Pivotal for Cucumber Mosaic Virus and Tobacco Rattle Virus Infections and Tolerance to Salt and Drought
Source: Int J Mol Sci. 2020 Mar 18;21(6):2083. doi: 10.3390/ijms21062083 (PMC7139328; doi:10.3390/ijms21062083)
Supplement: Supplementary file 1 [file ijms-21-02083-s001.zip › ijms-738733-Supplementary materials.docx]

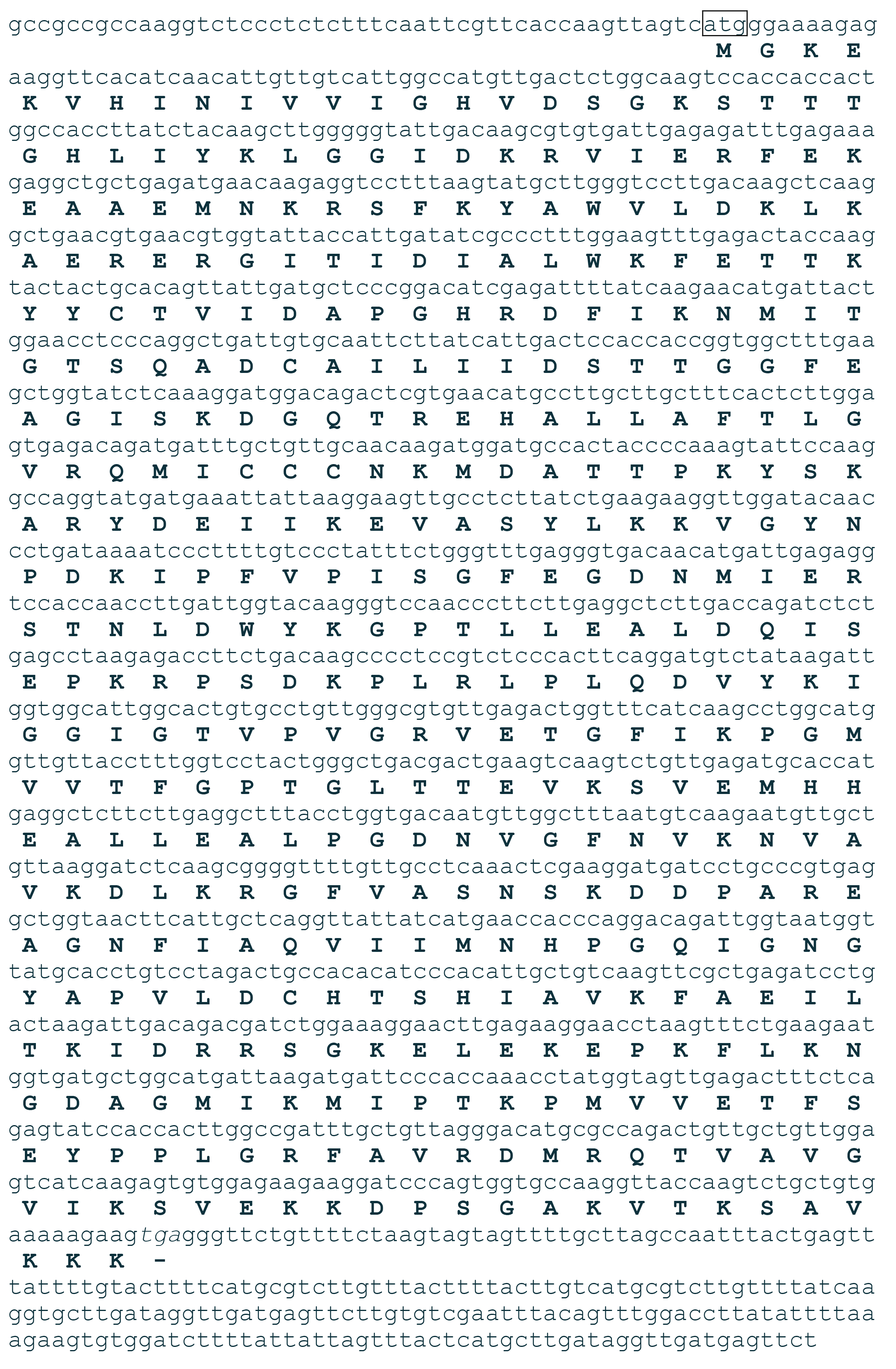


**Figure S1.** Full-length cDNA and deduced amino acid sequences of *LreEF1A4*. The 1,616-bp cDNA sequence of *LreEF1A4* harbors a 1,344-bp open reading frame region encoding a polypeptide of 447 amino acids. The amino acids are shown in boldface. The start and stop codons are marked with boxed and italic types, respectively.

>Full-length cDNA sequence of *LreEF1A4*

GCCGCCGCCAAGGTCTCCCTCTCTTTCAATTCGTTCACCAAGTTAGTC**ATG**GGAAAAGAGAAGGTTCACATCAACATTGTTGTCATTGGCCATGTTGACTCTGGCAAGTCCACCACCACTGGCCACCTTATCTACAAGCTTGGGGGTATTGACAAGCGTGTGATTGAGAGATTTGAGAAAGAGGCTGCTGAGATGAACAAGAGGTCCTTTAAGTATGCTTGGGTCCTTGACAAGCTCAAGGCTGAACGTGAACGTGGTATTACCATTGATATCGCCCTTTGGAAGTTTGAGACTACCAAGTACTACTGCACAGTTATTGATGCTCCCGGACATCGAGATTTTATCAAGAACATGATTACTGGAACCTCCCAGGCTGATTGTGCAATTCTTATCATTGACTCCACCACCGGTGGCTTTGAAGCTGGTATCTCAAAGGATGGACAGACTCGTGAACATGCCTTGCTTGCTTTCACTCTTGGAGTGAGACAGATGATTTGCTGTTGCAACAAGATGGATGCCACTACCCCAAAGTATTCCAAGGCCAGGTATGATGAAATTATTAAGGAAGTTGCCTCTTATCTGAAGAAGGTTGGATACAACCCTGATAAAATCCCTTTTGTCCCTATTTCTGGGTTTGAGGGTGACAACATGATTGAGAGGTCCACCAACCTTGATTGGTACAAGGGTCCAACCCTTCTTGAGGCTCTTGACCAGATCTCTGAGCCTAAGAGACCTTCTGACAAGCCCCTCCGTCTCCCACTTCAGGATGTCTATAAGATTGGTGGCATTGGCACTGTGCCTGTTGGGCGTGTTGAGACTGGTTTCATCAAGCCTGGCATGGTTGTTACCTTTGGTCCTACTGGGCTGACGACTGAAGTCAAGTCTGTTGAGATGCACCATGAGGCTCTTCTTGAGGCTTTACCTGGTGACAATGTTGGCTTTAATGTCAAGAATGTTGCTGTTAAGGATCTCAAGCGGGGTTTTGTTGCCTCAAACTCGAAGGATGATCCTGCCCGTGAGGCTGGTAACTTCATTGCTCAGGTTATTATCATGAACCACCCAGGACAGATTGGTAATGGTTATGCACCTGTCCTAGACTGCCACACATCCCACATTGCTGTCAAGTTCGCTGAGATCCTGACTAAGATTGACAGACGATCTGGAAAGGAACTTGAGAAGGAACCTAAGTTTCTGAAGAATGGTGATGCTGGCATGATTAAGATGATTCCCACCAAACCTATGGTAGTTGAGACTTTCTCAGAGTATCCACCACTTGGCCGATTTGCTGTTAGGGACATGCGCCAGACTGTTGCTGTTGGAGTCATCAAGAGTGTGGAGAAGAAGGATCCCAGTGGTGCCAAGGTTACCAAGTCTGCTGTGAAAAAGAAG***TGA***GGGTTCTGTTTTCTAAGTAGTAGTTTTGCTTAGCCAATTTACTGAGTTTATTTTGTACTTTTCATGCGTCTTGTTTACTTTTACTTGTCATGCGTCTTGTTTTATCAAGGTGCTTGATAGGTTGATGAGTTCTTGTGTCGAATTTACAGTTTGGACCTTATATTTTAAAGAAGTGTGGATCTTTTATTATTAGTTTACTCATGCTTGATAGGTTGATGAGTTCT

>Full-length amino acid sequence of LreEF1A4

MGKEKVHINIVVIGHVDSGKSTTTGHLIYKLGGIDKRVIERFEKEAAEMNKRSFKYAWVLDKLKAERERGITIDIALWKFETTKYYCTVIDAPGHRDFIKNMITGTSQADCAILIIDSTTGGFEAGISKDGQTREHALLAFTLGVRQMICCCNKMDATTPKYSKARYDEIIKEVASYLKKVGYNPDKIPFVPISGFEGDNMIERSTNLDWYKGPTLLEALDQISEPKRPSDKPLRLPLQDVYKIGGIGTVPVGRVETGFIKPGMVVTFGPTGLTTEVKSVEMHHEALLEALPGDNVGFNVKNVAVKDLKRGFVASNSKDDPAREAGNFIAQVIIMNHPGQIGNGYAPVLDCHTSHIAVKFAEILTKIDRRSGKELEKEPKFLKNGDAGMIKMIPTKPMVVETFSEYPPLGRFAVRDMRQTVAVGVIKSVEKKDPSGAKVTKSAVKKK

**Figure S2.** FASTA-formatted cDNA and deduced amino acid sequences of *LreEF1A4*. The start codon of *LreEF1A4* coding sequence is boxed in boldface, and its stop codon is indicated in bold italics.


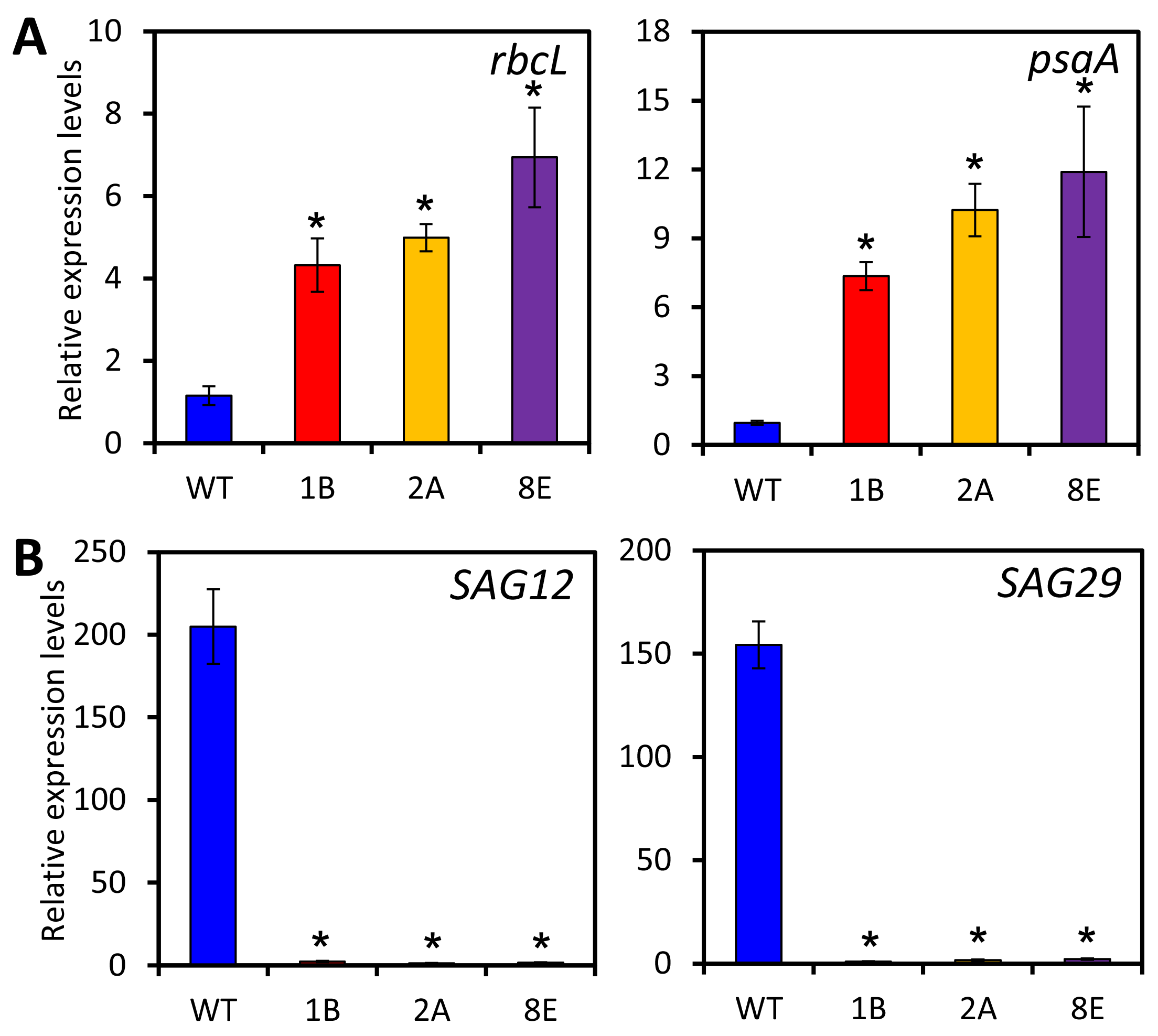


**Figure S3.** Expression levels of genes related with photosynthesis and senescence in wild-type and *LreEF1A4*-overexpressing transgenic petunia plants. Quantitative real-time polymerase chain reaction (PCR) analysis of transcript levels of photosynthesis-related genes (*rbcL* and *psaA*) in the leaves (**A**) and senescence-associated genes (*SAG12* and *SAG29*) in the flowers (**B**) of wild-type (WT) and *LreEF1A4*-overexpressing lines. The bottom leaves of 8-week-old plants and flowers at 8 days after anthesis were collected for expression assessment. Transcript levels were normalized to *26S rRNA*. Error bars represent standard error (SE) of the mean from three independent biological replicates. Asterisks denote significance of difference as determined by Student’s *t*-test (*p* < 0.05).


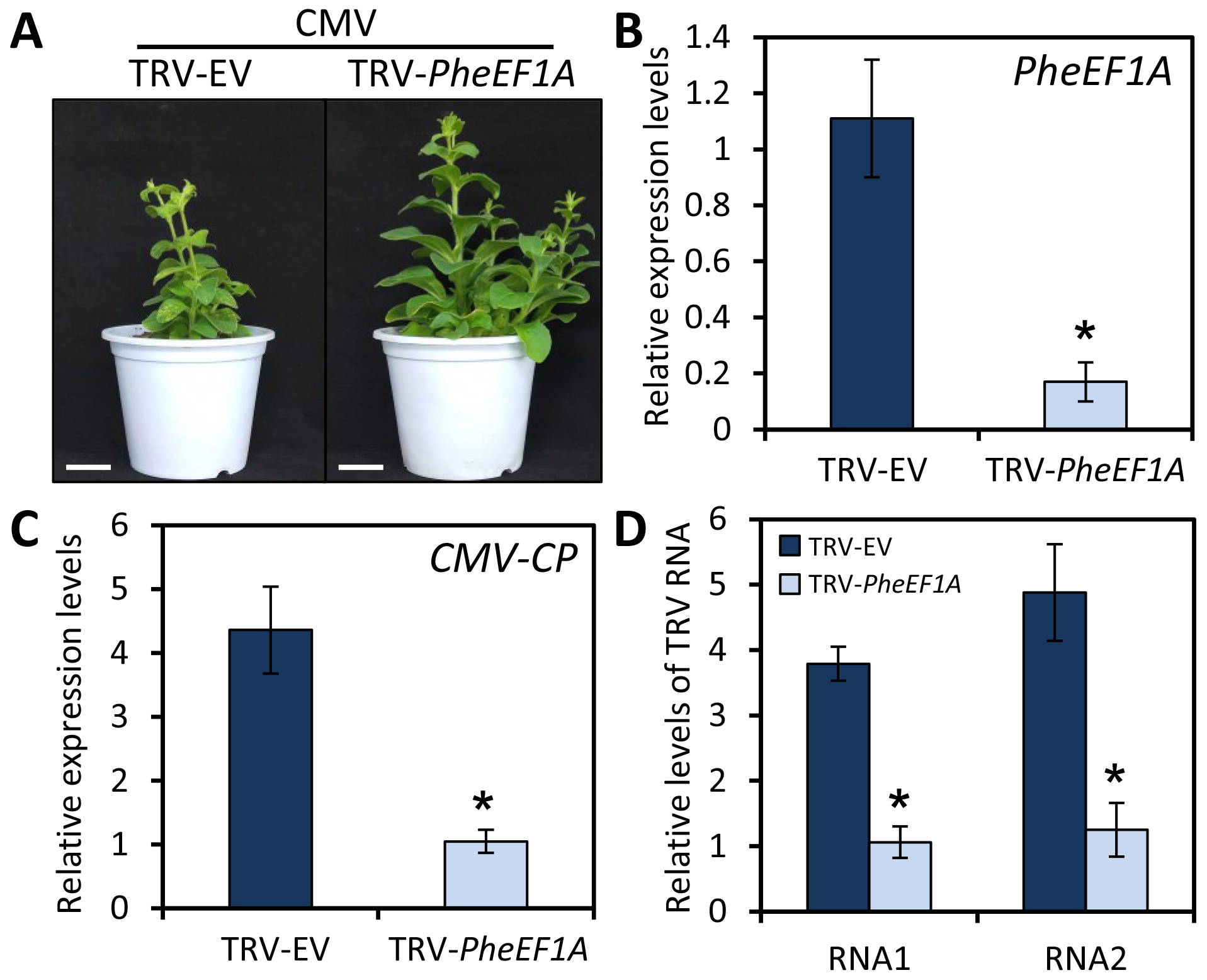


**Figure S4.** Reduced accumulation of cucumber mosaic virus (CMV) and tobacco rattle virus (TRV) in *PheEF1A*-silenced petunia plants by VIGS. (**A**) Disease symptoms of TRV empty vector (TRV-EV)- and TRV-*PheEF1A*-infected wild-type petunia plants at 40 days post inoculation (DPI) with CMV. Scale bars = 3.0 cm. Quantitative real-time polymerase chain reaction (PCR) analysis of *PheEF1A* (**B**), CMV coat protein (*CMV-CP*) (**C**), and TRV RNA1/RNA2 (**D**) transcript levels in uppermost leaves of TRV-EV- and TRV-*PheEF1A*-infected petunia plants at 40 DPI with CMV. *26S rRNA* was used as a reference gene. Error bars represent standard error (SE) of the mean from three independent biological replicates. Statistical difference was determined by Student’s *t*-test at *p* < 0.05 and marked by asterisks.

**
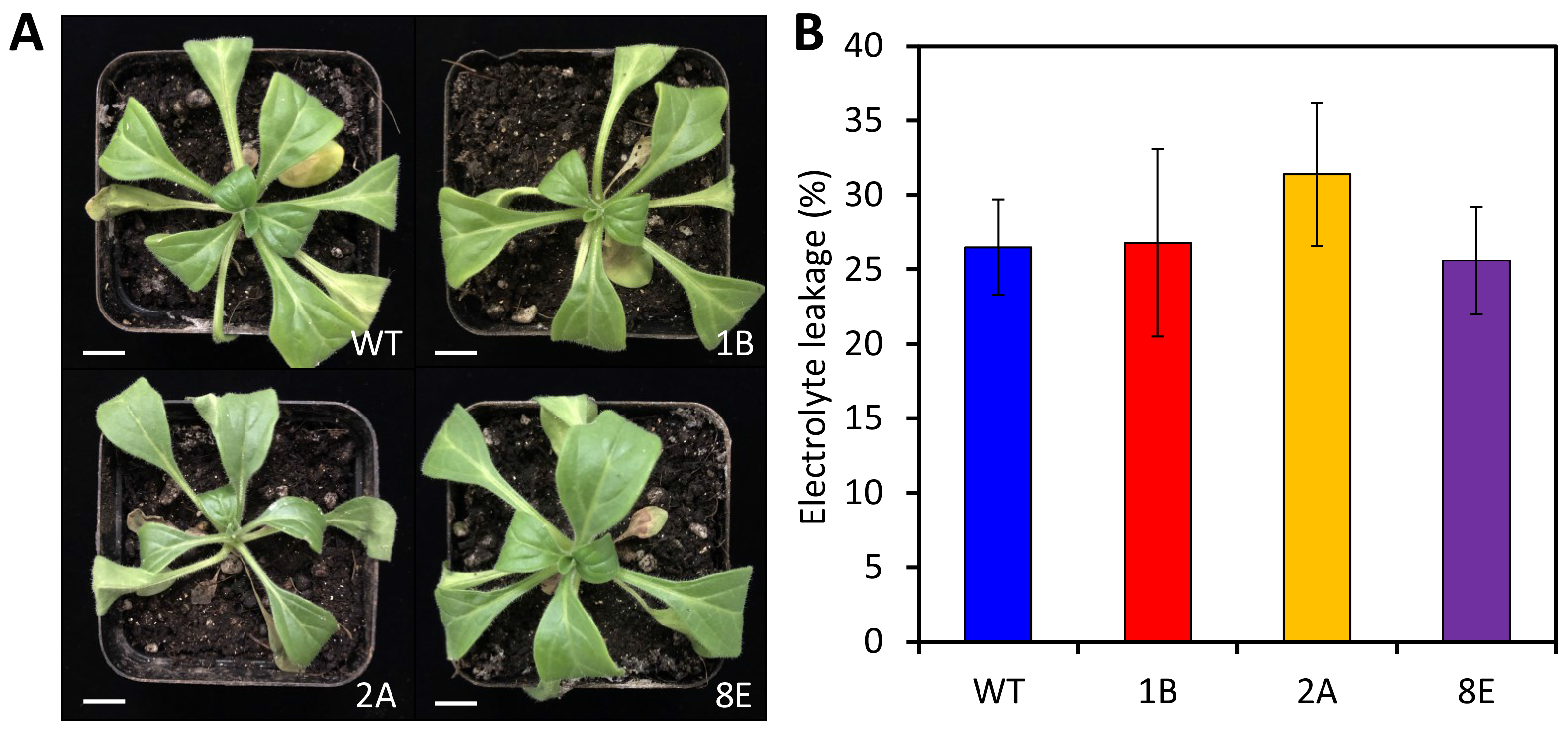
**

**Figure S5.** The impact of *LreEF1A4* overexpression on cold tolerance of transgenic petunia plants. Representative phenotypes (**A**) and electrolyte leakage (**B**) of wild-type (WT) and transgenic petunia seedlings (1B, 2A, and 8E) after treatment with 48 h of 4 ^o^C and subsequent 24 h of −2 ^o^C. Five-week-old seedlings were used for the treatment. Scale bars = 1.0 cm. Error bars represent standard error (SE) of the mean from three independent biological replicates. Statistical difference was analyzed by Student’s *t*-test at *p* < 0.05.

**Table S1.** Primers used for fragment amplification, semi-quantitative reverse transcription-polymerase chain reaction (RT-PCR), and quantitative real-time PCR.

| **Gene ID** | **Forward primer (5’-3’)** | **Reverse primer (5’-3’)** | **Product size** |
| --- | --- | --- | --- |
| For full-length amplification of *LreEF1A*s | | | |
| *LreEF1A1* (F1, R1) | CAAGTTTTTAGTTGAGTTCTGTC | ACTCGGACATAAATAGAAGAACG | 1,395 bp |
| *LreEF1A2* (F1, R1) | CGTTTTTGACTCCACCTAATATC | CCAACAAACGAGGGGATTCCTCG | 1,396 bp |
| *LreEF1A3* (F1, R1) | ATCAGCGACCGCAAGAGCCGCCG | GAAATCCCTTCGATTCATTTCTT | 1,392 bp |
| For overexpression construct of *LreEF1A4* | | | |
| *LreEF1A4* (F1, R1) | ATGGTACCATGGGAAAAGAGAAGGTTCA | ATGTCGACGGCTAAGCAAAACTACTACT | 1,395 bp |
| For gene expression analysis | | | |
| *LreEF1A4* (F2, R2) | CCAAACCTATGGTAGTTGAGACT | GGCTAAGCAAAACTACTACTTAG | 196 bp |
| *LreEF1A1* (F2, R2) | TATCAAGAAGAAGTGAGGCGTTC | GACCAAATAGAGCAGTAAAGCAAA | 154bp |
| *LreEF1A2* (F2, R2) | TCAAGAATGTGGAGAAGAAGGAA | CTGACAGAAATAGCACACAGACA | 182 bp |
| *LreEF1A3* (F2, R2) | AGAAGAAGGATCCCACTGGAG | GACGATCAAGCACCCAGTTCT | 186 bp |
| *CMV*-*CP* | ACAAATCTGAATCAACCAGTGCT | ACGAAGGTTGGGTGGTTAATAGT | 172 bp |
| TRV RNA1 | CAGTCTATACACAGAAACAGA | GACGTGTGTACTCAAGGGTT | 463 bp |
| TRV RNA2 | GGTTACTAGCGGCACTGAATAGA | TAGTACTCCCTTGGTTCGTCGTA | 225 bp |
| *rbcL* | ACTTACCAGCCTTGATCGTTACA | CGAGGCTTACACCTCCCTATTAC | 291 bp |
| *psaA* | TTCAAGGAGTTGGAGCTTGATAG | GGTTTGTATGGCTCACTCTGTTC | 206 bp |
| *SAG12* | CATGGAGAGGATGAAGGTTGTAG | AACACCACTGGAATAGAACTGGA | 279 bp |
| *SAG29* | TCTCAAGGGAAATAATGGGATTT | GGCGAGTAGATATGTCAGGATTG | 187 bp |
| *LrGAPDH* | TACATCTGGTGTGGTTTGTTGAG | AAAGCCAATTCATTGATAAAGCA | 178bp |
| *26S rRNA* | AGCTCGTTTGATTCTGATTTCCAG | GATAGGAAGAGCCGACATCGAAGG | 185bp |
| For inserted fragment amplification and expression analysis in VIGS assay | | | |
| *PheEF1A* (F1, R1) | CAAGGCTAGGTACGATGAAATTG | AAGTCACAACCATACCAGGCTTA | 314 bp |
| *PheEF1A* (F2, R2) | TTAGGCAACATGGGTAAAGAGAA | AAACTTCCACAAGGCAATATCAA | 249 bp |
